# Detection of Cardiovascular Disease from Clinical Parameters Using a One-Dimensional Convolutional Neural Network

**DOI:** 10.3390/bioengineering10070796

**Published:** 2023-07-03

**Authors:** Mohammad Mahbubur Rahman Khan Mamun, Tarek Elfouly

**Affiliations:** Department of Electrical and Computer Engineering, Tennessee Technological University, Cookeville, TN 38505, USA; telfouly@tntech.edu

**Keywords:** heart disease, artificial intelligence, 1D CNN, diagnosis, feature selection

## Abstract

Heart disease is a significant public health problem, and early detection is crucial for effective treatment and management. Conventional and noninvasive techniques are cumbersome, time-consuming, inconvenient, expensive, and unsuitable for frequent measurement or diagnosis. With the advance of artificial intelligence (AI), new invasive techniques emerging in research are detecting heart conditions using machine learning (ML) and deep learning (DL). Machine learning models have been used with the publicly available dataset from the internet about heart health; in contrast, deep learning techniques have recently been applied to analyze electrocardiograms (ECG) or similar vital data to detect heart diseases. Significant limitations of these datasets are their small size regarding the number of patients and features and the fact that many are imbalanced datasets. Furthermore, the trained models must be more reliable and accurate in medical settings. This study proposes a hybrid one-dimensional convolutional neural network (1D CNN), which uses a large dataset accumulated from online survey data and selected features using feature selection algorithms. The 1D CNN proved to show better accuracy compared to contemporary machine learning algorithms and artificial neural networks. The non-coronary heart disease (no-CHD) and CHD validation data showed an accuracy of 80.1% and 76.9%, respectively. The model was compared with an artificial neural network, random forest, AdaBoost, and a support vector machine. Overall, 1D CNN proved to show better performance in terms of accuracy, false negative rates, and false positive rates. Similar strategies were applied for four more heart conditions, and the analysis proved that using the hybrid 1D CNN produced better accuracy.

## 1. Introduction

Among all the chronic diseases in the world, heart disease is regarded as one of the most alarming. Typical heart disease occurs because of a lack of blood supply to body parts, including the heart itself [1]. Additionally, due to obstacles in coronary arteries or their narrowing, the blood flow slows down, and heart failure happens [2]. Some heart disease symptoms are dizziness, shortness of breath, swollen limbs, physical weakness, chest pain, etc. [3]. Although the main underlying reason for heart disease is atherosclerosis, which is plaque building in arteries, this buildup starts early in life. However, the symptoms usually do not appear until the person is the age of around 50 or higher [4]. From 2010 to 2020, approximately 18.7% more deaths occurred due to CVD issues, with the number of deaths increasing to around 19 million [5]. According to the World Health Organization (WHO), about 17.90 million people globally died from cardiovascular disease in 2016 [6]. The European Society of Cardiology (ESC) published a report in which it was estimated approximately 3.8 million people were identified with heart disease yearly. Within that population, 50% of patients die within the first 1 to 3 years [7]. Besides negligence, many diagnosed CVD patients cannot receive proper treatment due to financial challenges.. The following subsections discuss the recent research progress in heart disease diagnosis, specifically focusing on using deep learning. Afterward, the research goal of this manuscript is addressed. 

**Figure 1:**
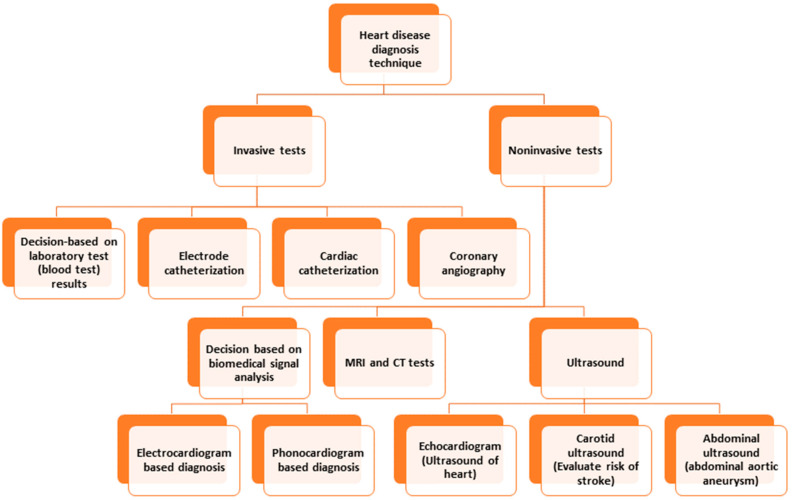
Heart disease diagnosis techniques.

Conventional invasive methods for heart disease detection depend on laboratory tests, physical tests, investigation by a physician, etc. [8]. Among the invasive techniques, angiography and catheterization are prominent. Catheterization is a medical procedure in which a thin, flexible tube called a catheter is inserted into a blood vessel to diagnose and treat heart conditions. Angiography is a medical imaging technique that uses X-rays and a contrast agent to visualize the blood vessels inside the body. Both methods suffer from limitations, such as invasiveness (risking bleeding or infection), limited access, expensiveness, or limited visualization, which hinders obtaining a scenario of a specific time, etc. [9,10]. Computed Tomography (CT) and Magnetic Resonance Imaging (MRI) are commonly used to detect and diagnose heart disease. Both modalities have limitations, which include requiring the patient to be still in a particular position, exposure to ionizing radiation, expensive procedures, inability to detect mild or early-stage heart disease, etc. [11,12]. The limitations of ultrasound to detect heart disease are limited resolution, failure to detect minor abnormalities, lack of ability to penetrate through bone or dense tissues, need for a skillful operator, limited use for patients with pacemakers, etc. [13]. Figure 1 depicts the different diagnostic techniques for heart disease. 

**Figure 2:**
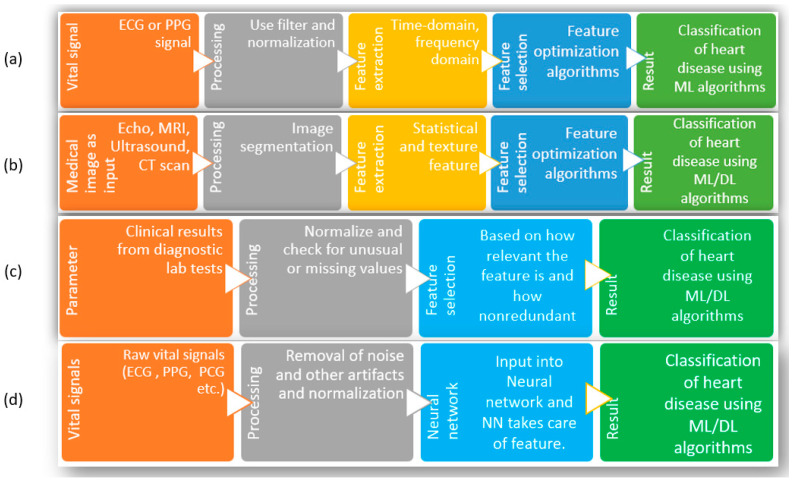
Different methods of heart disease classification using ML and DL. (**a**) Input as vital signs and extraction of features, (**b**) input as images (Echo, MRI, ultrasound, CT scan), (**c**) input as clinical parameters, (**d**) input as raw vital signs.

To solve the problems related to the existing noninvasive methods to identify heart diseases, researchers have attempted to use the advancements in machine learning (ML) and deep learning (DL) combined with easily acquirable vital signs such as an electrocardiogram (ECG), photoplethysmography (PPG), phonocardiogram (PCG), etc. [14]. To elaborate, the reasons behind choosing ML and DL are as follows. First, many popular ML and DL algorithms are now available for use, which already have applications across different types of biomedical data, such as clinical, genetic, imaging, biochemical, etc., to understand diseases, develop new treatments, and improve patient care [15,16,17,18]. Second, with the advancement of sensor technology, the acquisition of ECG, PPG, and PCG is becoming more accessible than before. Third, compared to the manual observation of test results, physical checkups, diagnostic tests, etc., ML and DL can dig up feature contributions that may be hard for humans to comprehend. As shown in Figure 2, the typical attempts using ML and DL with clinical data or biomedical signals to detect heart disease can be divided into four groups. Since researchers in this regard have performed numerous studies, the whole research area is divided into several groups and discussed here to summarize the development adequately. Group-(a) considers biomedical signals such as ECG or PPG [19,20,21,22,23] as inputs. Since these signals are easy to acquire and smaller sensors are available with a powerful capacity to process these signals, these are used as input directly. After the information is obtained, the signals are preprocessed for noise cancellation and normalization [24,25]. 

With the preprocessed signal, the necessary features are extracted; depending on the algorithm, the features are from the time domain, the frequency domain, or a combination of both domains [24,26,27,28,29,30,31]. With all the extracted features, optimization of features is attempted. Optimization is necessary to ensure all the finalized features are relevant and nonredundant. Finally, those optimized features are fed into the ML algorithms to obtain classification of heart disease. The limitations facing this group of studies are that, since the method strictly depends on biomedical signals (such as ECG/PPG/PCG), it requires other supplementary information to diagnose correctly. In addition, the extraction of features depends on researchers, and there are no central or general guidelines for signal acquisition or the number or types of features used in the algorithms. Because of these, it is not easy to compare or combine results among experiments carried out in different studies. The studies in group-(b) start with medical images as inputs to the process. Similar to the steps in group-(a), this method moves through preprocessing (filtering for noise, smoothing of images), feature extraction and selection, taking care of the part where the image is cropped or selected, and texture/statistical features are selected. Finally, those features are used with ML/DL algorithms to classify different heart conditions and diseases [32,33,34,35,36,37]. Since many high-quality images are required to train these models, the availability of such large datasets remains the main challenge facing researchers. As well, the images from new patients need to maintain similar quality for the algorithm to perform with fewer false results. Additionally, manual feature extraction has similar problems as in group-(a); these studies must follow standardized guidelines to compare performance consistently. Due to the availability of several datasets with clinical parameters, the studies in group-(c) are prevalent among scientists [38,39,40,41,42,43,44,45,46,47,48,49,50]. Here, a specific list of clinical parameters is used as input for the system; the number and types of parameters depend on which dataset is selected. After removing the outliers and filling up the missing values, the exclusive features are optimized using feature selection algorithms. The advantage of these studies is that the clinical parameters are strictly measured in the case of a heart disease patient; thus, they are already significant enough to be included. On the downside, nearly all the studies are based on online databases.

In contrast, most datasets were not created for this purpose. Since feature numbers cannot be increased or modified, starting with a dataset having a significantly high number of features that are not only relevant but also nonredundant is essential [51]. In group-(d), the input in the figure consists of biomedical signals. However, biomedical information can be used here, such as clinical information, images from X-rays, ultrasound, CT scans, MRI, etc., so any of these can be the starting point. In preprocessing steps, the information is filtered/normalized/cleaned/smoothed for further use. Then, that information is used as input for neural networks, and the neural network decides the weights of each input and subsequent neuron nodes to produce classification results [38,52,53,54,55,56,57,58,59,60,61,62]. The general limitations of this group are the same as the previous group of studies in the case of images or biomedical signals as a starting point. On top of those limitations, the neural network hyperparameters must be tuned perfectly for the model to be used for another dataset. In the case of tabular format data (clinical parameters), a change in the chronology of the parameters in the input should not change the outcome, which is a significant limitation of these studies. Further, similar to the excellent performance of 2D convolutional neural networks with feature extraction and classification for image input, 1D CNN has been quite popular among researchers for biomedical data, such as ECG, PPG, PCG, etc., for the classification of a heart condition such as arrhythmia [63,64,65,66]. A significant advantage of using a 1D CNN for biomedical data is that it can learn local patterns in the data and extract features which are relevant to the task at hand [67]. For biomedical data, whether they are similar to ECG, which is sequential and time series data, or image type, such as X-ray or MRI, the use of 1D/2D CNN is practical due to the ability of the CNN to extract local patterns. However, to take advantage of 1D CNN for nonsequential tabular data with no time-specific information, the main challenge is to ensure the model performance is consistent, irrespective of feature order in the tabular data. There are several areas for improvement in the use of machine learning and deep learning techniques for detecting heart disease, as discussed above. These include increasing the size and diversity of the dataset, addressing imbalances in the dataset, selecting, and optimizing the number of features, selecting the appropriate model and fine-tuning its hyperparameters, and expanding research to cover a broader range of heart conditions.

This work presents an intelligent decision-making model for diagnosing various types of heart diseases using a 1-dimensional convolutional neural network. A dataset was compiled from multiple records to accomplish this, and the data imbalance issue was addressed through undersampling. The modified 1D CNN was then applied to the tabular data, regardless of chronology. The results were compared to those from other contemporary machine learning models. All computations and processing were conducted using Google Colab and Python programming, including the TensorFlow framework. The main contributions of this study are as follows:

A. Proposing a heart disease diagnosis model using the 1D CNN, making use of a large dataset with clinical parameters;
B. Presenting the analysis of 1D CNN while dealing with dataset imbalance;
C. Comparing with contemporary ML algorithms, using performance evaluation metrics;
D. Making recommendations, including tuned hyperparameters for 1D CNN and featured optimization algorithms for developing a system to diagnose heart disease in its early stages.

The model can help medical practitioners provide early and appropriate medical therapies by properly detecting many forms of cardiac problems. Better treatment strategies, better patient outcomes, and, perhaps, even cheaper healthcare expenditures can result from this. The model’s capacity for intelligent decision-making offers the possibility of early diagnosis of heart problems, enabling people to take preventative measures to manage their cardiovascular health. The model’s main contribution is its capacity to increase healthcare quality, which will result in a healthier population and greater overall wellbeing. The paper is structured as follows. Section 2 discusses the proposed technique in detail. In Section 3, the data analysis using the proposed method is described. Section 4 presents the results and performance analysis, and Section 5 discusses the overall experiments and limitations of the research. The paper concludes in Section 6.

## 2. Proposed Technique

**Figure 3:**
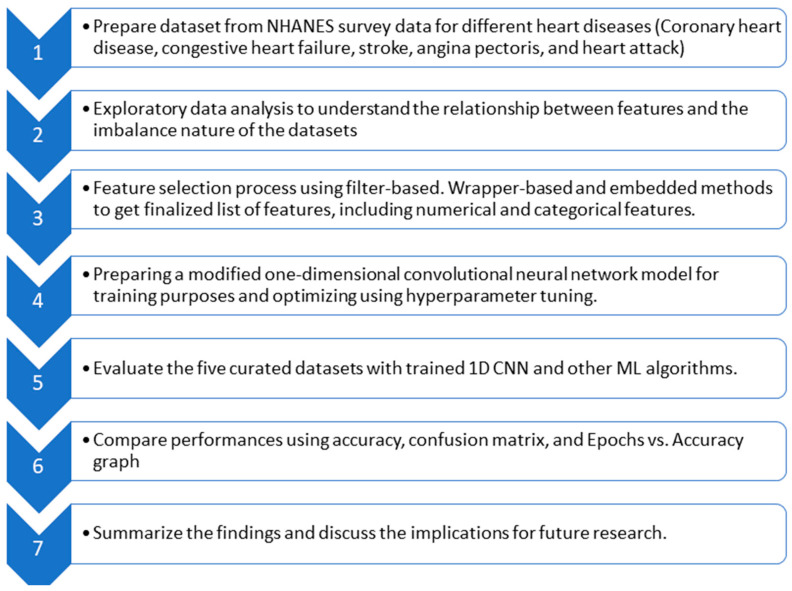
Flow diagram of the whole experiment.

In this section, the proposed technique is discussed. After introducing the dataset and bringing forth the imbalance in data, feature optimization is elaborated. Then, the model based on a convolutional neural network is proposed to diagnose heart disease. Figure 3 provides a flow diagram showing the general idea behind the whole technique. 

### 2.1. Dataset Selection and Preparation

Among the renowned research in the field of heart disease detection, the datasets which have been used most often are the Cleveland dataset [68], UCI repository dataset [69], MIT–BIH dataset [70], several versions of the MIMIC dataset [71,72], etc. Among those, the Cleveland dataset and UCI repository constitute clinical parameters. However, the frequently used heart disease detection datasets have few records. The MIT–BIH dataset is used mainly for diagnosing arrhythmia, since no other class information is available and the number of samples is minimal. The only popular dataset with a large amount of data is the MIMIC dataset and its different versions. MIMIC, MIMIC II, and MIMIC III have patient biosignals such as ECG, PPG, and ABP. Although those datasets are appropriate for diagnosing issues for which no other clinical parameters are required, due to a lack of class information about heart disease, these datasets could not be more helpful in detecting specific heart diseases. 

In this study, the database chosen was from the National Health and Nutrition Examination Survey by CDC [73]. The reasons behind the choice are that it is a large dataset with a significantly higher number of relevant clinical parameters covering a wide range of periods. Although the dataset was not available for input for the model, it is possible to accumulate the data from several years and surveys. The dataset used in this study was prepared by compiling NHANES data from 1999–2000 to 2017–2018. In NHANES, different categories of data are stored in different sections of the database; for heart disease diagnosis, the sections used are demographic, examination, laboratory, and questionnaire data. Since the datasets involved both numerical responses as well as categorical responses, there needed to be refinement in terms of eligibility for heart disease diagnosis and preparation for input to the algorithm for training. The patient’s record of heart diseases such as coronary heart disease, stroke, heart attack, angina pectoris, and congestive heart disease was used as a class variable for any ML or DL technique. 

For example, the data documentation format from the section “examination” is shown in Table 1. Here, 2017–2018 data documentation has been picked from the NHANES database.

**Figure 4:**
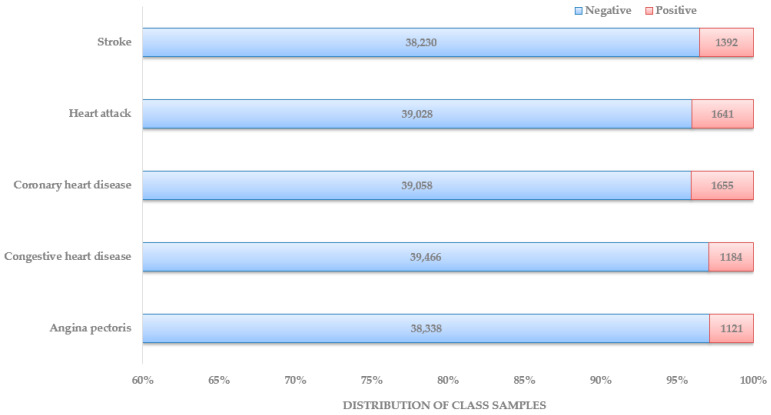
Distribution of patients by class.

These data were published in February 2020, focusing on three consecutive blood pressure measurements and heart rate/pulse to obtain accurate BP numbers. Each data documentation has a detailed description of the eligible sample, protocol and procedure, and data processing and editing. Unlike the other datasets used in recent research articles, this makes the NHANES the most organized and systematic collection of data that can be used for heart disease diagnosis. In Figure 4, the distribution of patient class is depicted. The dataset preparation process started with locating NHANES survey data from the CDC website from 1999–2000 years until 2017–2018 years. Under each year, data sections were named: demographics data, dietary data, examination data, laboratory data, and questionnaire data. Based on the responses found about the presence and type of heart disease, five separate datasets were prepared, one for each heart disease: stroke, heart attack, coronary heart disease, congestive heart disease, and angina pectoris. 

Data preparation involves methods such as data cleaning, data normalization, data encoding, data transformation, data imputation, etc. The necessity of all those methods or a subset of those depends on the dataset type and objective of the study. After collecting the dataset from NHANES survey data as discussed before, the features were ‘SEQN’, ‘Gender’, ‘Age’, ‘Annual-Family-Income’, ‘Ratio-Family-Income-Poverty’, ‘X60-sec-pulse’, ‘Systolic’, ‘Diastolic’, ‘Weight’, ‘Height’, ‘Body-Mass-Index’, ‘White-Blood-Cells’, ‘Lymphocyte’, ‘Monocyte’, ‘Eosinophils’, ‘Basophils’, ‘Red-Blood-Cells’, ‘Hemoglobin’, ‘Mean-Cell-Vol’, ‘Mean-Cell-Hgb-Conc.’, ‘Mean-cell-Hemoglobin’, ‘Platelet-count’, ‘Mean-Platelet-Vol’, ‘Segmented-Neutrophils’, ‘Hematocrit’, ‘Red-Cell-Distribution-Width’, ‘Albumin’, ‘ALP’, ‘AST’, ‘ALT’, ‘Cholesterol’, ‘Creatinine’, ‘Glucose’, ‘GGT’, ‘Iron’, ‘LDH’, ‘Phosphorus’, ‘Bilirubin’, ‘Protein’, ‘Uric Acid’, ‘Triglycerides’, ‘Total-Cholesterol’, ‘HDL’, ‘Glycohemoglobin’, ‘Vigorous-work’, ‘Moderate-work’, ‘Health-Insurance’, ‘Diabetes’, ‘Blood-Rel-Diabetes’, and ‘Blood-Rel-Stroke’. As part of data preparation, the first step was to drop the features that were not directly related or would not have any impact on the training model. The following columns from the dataset were dropped: ‘SEQN’, ‘Annual-Family-Income’, ‘Height’, ‘Ratio-Family-Income-Poverty’, ‘Health-Insurance’, ‘Lymphocyte’, ‘Monocyte’, ‘Eosinophils’, ‘Mean-Cell-Vol’, ‘Mean-Cell-Hgb-Conc.’, ‘Hematocrit’, and ‘Segmented-Neutrophils.’ Since not all the features were numerical, the categorical data were transformed into numerical using one hot encoding method. The affected features were ‘Gender (male/female)’, ‘Diabetes (Yes/No)’, ‘Blood-Rel-Diabetes (Yes/No)’, ‘Blood-Rel-Stroke (Yes/No)’, ‘Vigorous-work (amount of V. work)’, and ‘Moderate-work (amount of M. work)’. After that, the data normalization method was applied to make sure that features with larger values did not dominate the ML algorithm. A significant issue that required attention was the imbalanced state of available datasets, as shown in Figure 4. Since all the studies with a substantial number of features or parameters have used datasets available from medical centers or online, those datasets need to be balanced in many cases. With the current constraint of the imbalanced dataset with medical data, which can be used for heart disease diagnosis, before feeding data into the classification model, techniques such as over-/undersampling, SMOTE, data augmentation, etc., can be used to minimize the effect [63,64,65]. In this dataset, the method with the different class weights assigned to class labels was implemented to solve the data imbalance problem. 

### 2.2. Feature Selection 

Since the dataset used in this study was in the form of clinical parameters, the next step for the dataset was to select features that were not only relevant but also nonredundant. Feature selection methods can be divided into three categories [74,75]: filter-based, wrapper-based, and embedded-based. Filter-based feature selection ranks the features based on different statistical tests. The filter-based method is independent of a classification model, so there is no classifier bias due to no interaction with the classifier. However, there is also no chance of fine-tuning possible using the classifier for the same reason. Due to the advantages of interpretability and dimensionality reduction, the minimum Redundancy Maximum Relevance (mRMR) has been used as a filter-based feature selection [76,77]. 

The wrapper-based method considers the performance of the selected classifier algorithm using the feature in question. It shows better classification performance [77]. In this study, recursive feature elimination (RFE) was used from the aspect of the wrapper-based method. RFE was used for two main reasons: not only is the number of features to select not fixed, but there can also be any number of algorithms to choose from while not confined to one. These hyperparameters can be modified to obtain the optimum number of features for the best classifier [78]. 

In the embedded-based method, the classifier changes its hyperparameters or internal parameters to ensure the most effective weights in each feature are selected to achieve the maximum accuracy for any chosen performance metrics. As such, the feature selection step and model preparation happen in the same step in the embedded-based model, unlike the other two methods. Studies have shown that, with a higher number of features, the effectiveness of random forest to measure interactions among features accurately declines [79]. Additionally, the random forest does not automatically remove the redundant features, which hampers the model’s performance [80]. 

**Figure 5:**
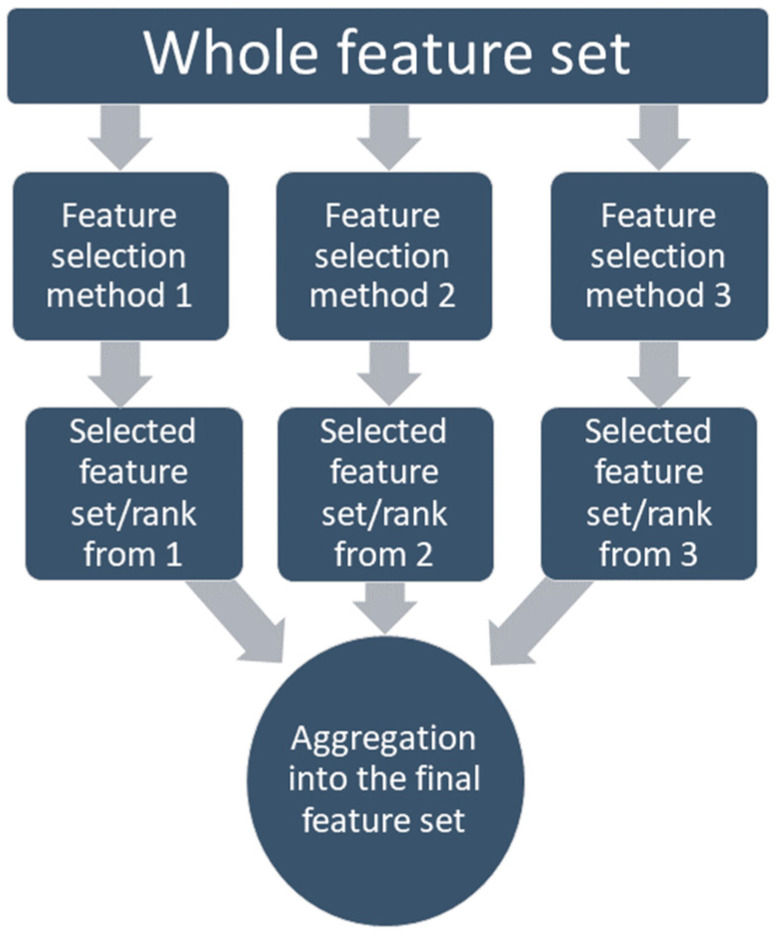
Aggregation-based ensemble feature selection method.

On the other hand, the regularization-based methods (lasso, ridge, elastic, etc.) use penalization. This can discard the redundant features (lasso) or decrease them to a minimal value (elastic); on the other hand, these still detect the interactions among features [81,82]. Many studies have compared the performance of the different methods with different classifiers and concluded that there is no such ‘perfect feature selection method’ for all problem types [75,83,84,85,86,87]. That is why the feature selection method which was chosen for this study is based on the ensemble method, where the feature selection methods are combined so the different strengths can be combined [88]. Several studies have proved that the ensemble-based feature selection method outperforms the single-feature selection methods if the computational load is not a concern [89,90,91]. Along with performance, the ensemble-based method also has proven to have more stability in the system by allowing a minor change in data, which means becoming more robust [92]. In this study, an aggregation-based ensemble method was applied, which allows combining the feature set achieved from different methods into one set through either union (liberal approach) or intersection (restrictive approach), or staying between those two using a threshold, as shown in Figure 5. 

### 2.3. Convolutional Neural Network

A 2D convolutional neural network (CNN) is a type of neural network designed explicitly for image-processing tasks. It is composed of multiple layers of artificial neurons that process and analyze images through the use of convolutional filters. The primary function of a CNN is to extract features from an input image and use these features to classify the image or make a prediction. A 2D CNN is called a “2D” network because it processes images in two dimensions, with both width and height. One of the key advantages of a 2D CNN is its ability to process images at different scales and orientations. Using multiple convolutional layers with different-sized filters, a CNN can learn to recognize features at different levels of abstraction, such as edges, shapes, and objects. A 2D convolutional neural network is a powerful tool for image processing tasks, such as object recognition and image classification. 

A 1D convolutional neural network (CNN) is a type of deep learning model designed to process one-dimensional data sequences, such as time series or text. It is a variant of the more general 2D CNN, which is designed to process two-dimensional data arrays such as images. One of the critical features of a 1D CNN is its ability to learn local patterns or features in the one-dimensional input data using a set of learnable convolutional filters sliding over the data. For one-dimensional data, the 1D CNN has several significant advantages. First, the computational complexity in 1D CNN is significantly lower than in 2D CNN. Second, 1D hidden layers use a shallow architecture format (usually around 10k parameters to be tuned). Third, with typical architecture, 1D CNN can be calculated using a standard computer. In contrast, for 2D CNN, the use of GPU is mandatory. Recent studies proved that with limited labeled data and high-variation 1D data, the 1D CNN showed superior performance [93,94,95,96,97,98,99,100,101,102,103,104,105]. 

In this study, the 1D CNN was used for structured data in a tabular format. However, those were not time series data, meaning no time information or dependencies were available. The typical use of 2D CNN is for image-based data, due to how CNN architecture works; furthermore, in this study, the motivational reasons behind the 1D CNN are, first, the same as for an image, which is a collection of pixel values with a limit in values, the tabular data can be kept within a limit using a normalization technique. Second, the position of pixels is critical in 2D CNN. Similarly, positioning features in input, which is tabular formatted data, is critical. Hence, finding the correct position for the features in the tabular list is challenging, which can be figured out using CNN architecture. Third, we used feature optimization in this study since medical data have to be rational. This helps the model to be more explainable. However, similar to 2D CNN, in 1D CNN, the architecture is in charge of whether a specific feature strongly correlates to the class variable or has a strong relationship with other features. 

A convolution filter is a mathematical operation applied to an input image to extract features or patterns from the image. A kernel, a small matrix of weights, defines the filter. When the kernel is applied to the input image, it slides over the image, performing a dot product between the entries in the kernel and the values of the pixels in the image at each position. The dot product is then used to compute a new value for the pixel, which is added to the output image. The two attributes of the input image used by a convolution filter are local connectivity and spatial locality. Local connectivity means that, when applied, each filter is only connected to a small input image area. This means that the filter can only consider a small region of the image at a time rather than the entire image. Spatial locality refers to the fact that the pixels affected by the filter tend to be spatially correlated, meaning they are likely to have similar values. This property allows the filter to extract meaningful patterns or features from the image. These characteristics help CNN to detect shapes or edges. Suppose this advantage of 2D CNN is tried to be replicated in 1D CNN. In that case, the problem arises that, unlike nearby pixels in the image, there may not be a local correlation among adjacent features. Moreover, another essential point to remember is that, for the 1D CNN model to be consistent, the change in the order of columns should not have any impact. 

**Figure 6:**
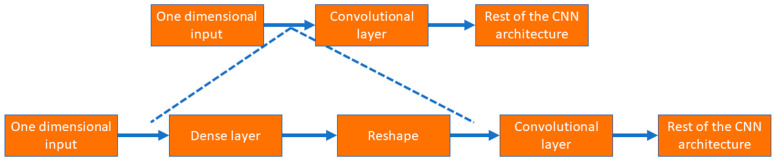
The process to use tabular data in 1D CNN architecture.

As shown in Figure 6, the input layer involves one-dimensional tabular format data; instead of putting those data into a convolutional layer, those data are initially fed to a dense layer with many nodes. The number of nodes in the dense layer is fixed, so they can be reshaped into multiple channels of one-dimensional data with lengths equal to s, i.e., the number of features. This modification allows the input data to be increased in number (dense layer) and rearranged (reshaped layer). Instead of the standard input data, the convolutional layer received complex patterns in the data in a multichannel format. This helps the convolutional filters to learn the non-linear mapping of complex feature combinations.

## 3. Data Analysis

In this section, the complete data analysis for the proposed study is elaborated. The section starts with how the data imbalance issue was dealt with and the preprocessing of the dataset to be used for feature optimization. The following subsection notes the ranked or selected features after using feature selection algorithms. Next, the 1D CNN architecture, which was used to train the model, is discussed regarding layer information, including hyperparameter tuning. 

### 3.1. Data Imbalance and Exploratory Analysis

After organizing and accumulating the NHANES dataset for the coronary heart disease (CHD) class variable, the next step was preparing the dataset for feature optimization or selection. While checking the dataset, one thing that stands out is the imbalance of the dataset. Regarding class variables, the number of no-CHD is much higher than that of CHD patients. In a recent literature survey, researchers found, in 49 prominent published articles, that the dataset used for heart disease diagnosis was imbalanced [106]. In this study, the algorithmic level was used, where the weight for the class variables can be changed based on the number of training instances in each class. The proper way is to try a series of different weight ratios to both the class variable and measure the performance matrices [107,108,109]. The best weight ratio is the optimum solution based on the result. Since, in the case of oversampling, many new samples have to be created that, although mathematically logical, are not from original data but rather created based on some rule which is based on original data; in this study, the impact of undersampling and weight assignment have been analyzed. 

**Figure 7:**
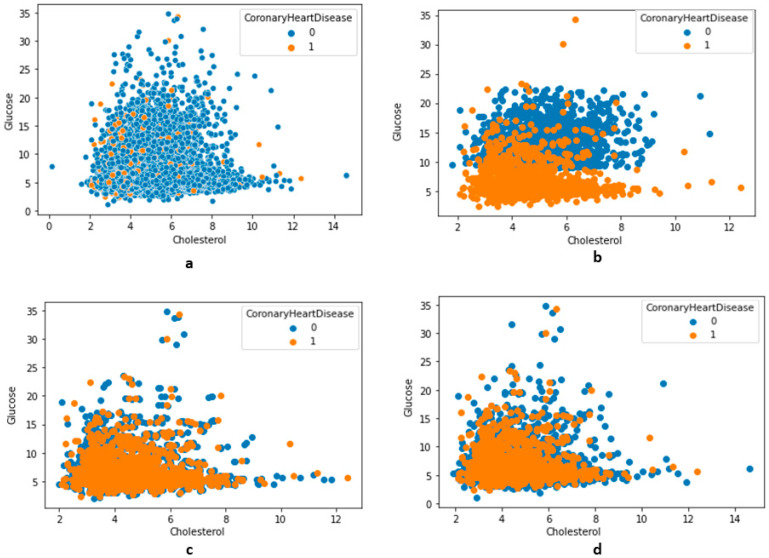
The distribution of features along with class variables (no-CHD—0, CHD—1). (**a**) Total distribution (no undersampling), (**b**) using undersampling NearMiss version 2, (**c**) using undersampling NearMiss version 3, (**d**) using an undersampling condensed nearest-neighbor.

The undersampling technique removes the sample from training data (the majority class) where the distribution in class variables is skewed, such as 1:10, 1:20, or even 1:2. The most straightforward undersampling technique in the calculation is removing samples randomly. This process is simple to execute; however, since the samples are removed without concern for the proximity of the decision boundary between the class variables, it is questionable. The two methods implemented in this study were the near-miss rule [110] and the condensed nearest-neighbor rule [111]. The latter technique has three versions, i.e., NearMiss versions 1, 2, and 3. Version 1 is where the sample is selected when it has the minimum average distance from the nearest three neighbors. Version 2 is where the algorithm selects the sample from the majority class with a minimum average distance from the three furthest samples from the minority class. In version 3, the samples from the majority class are selected individually to be closest to the minority class samples. The NearMiss algorithms were implemented in this study, as shown in Figure 7. Using the condensed nearest-neighbor method (CoNN), a variation of the undersampling technique to choose a subset of samples results in no loss in the model’s performance. Compared to the NearMiss techniques, the CoNN technique is prolonged. Considering the limitations of other resampling techniques and the results from undersampling techniques (in Figure 7), in this study, the number of majority classes was reduced to 1:3 from approximately 1:25. An approach that can be implemented to solve or mitigate the dataset imbalance issue is using weight-to-class variables. Different weights to no-CHD and CHD class variables were used during model training, and, based on performance metrics, the best weight was used. In this study, compared to the weight put on no-CHD, double weight was given to the CHD class variable. The ‘class_weight’ parameter was used to assign value for class variables.

**Figure 8:**
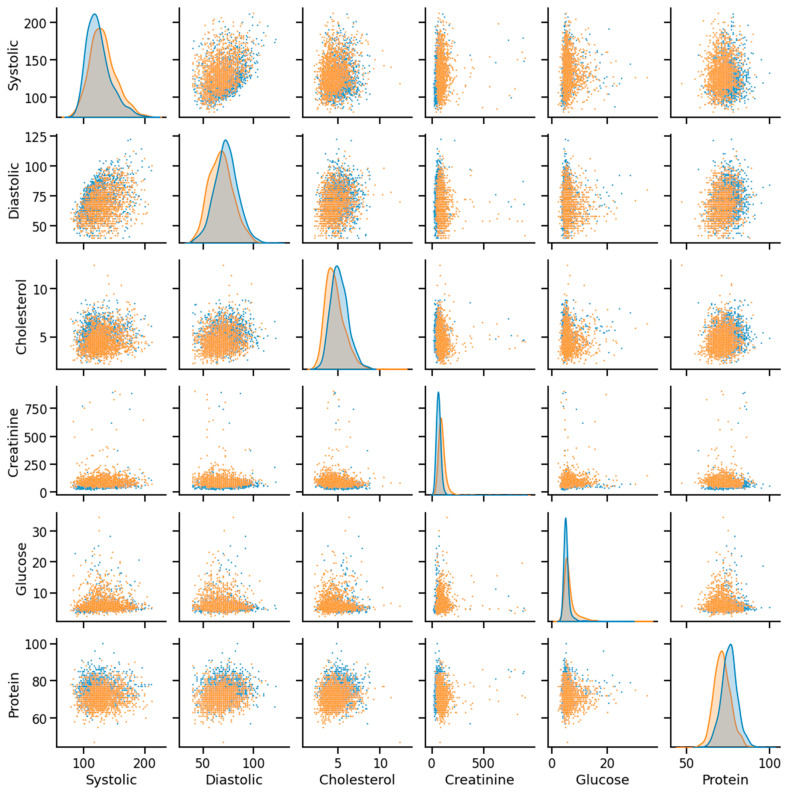
Example of data distribution (orange—CHD, blue—noCHD).

**Figure 9:**
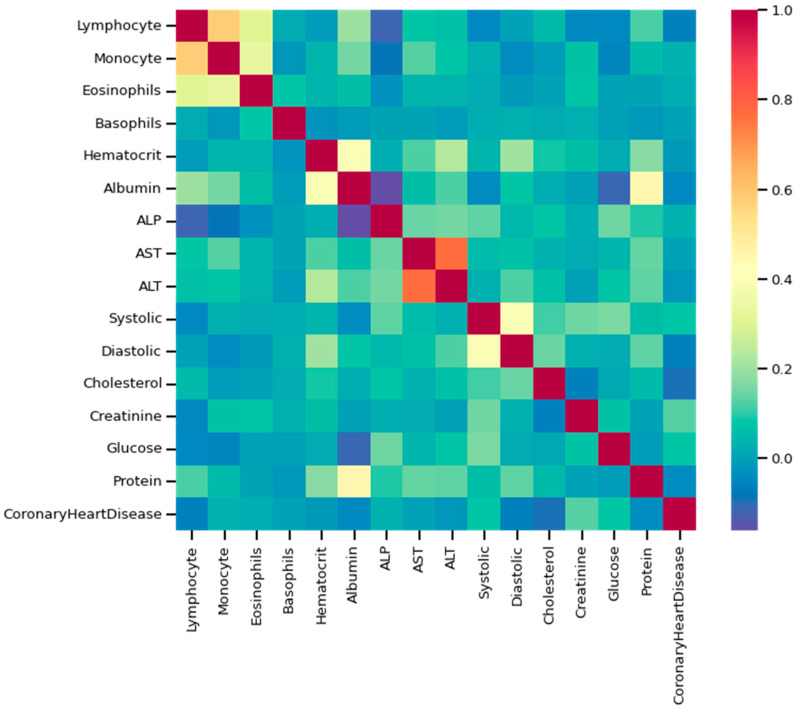
Heatmap using some of the features and class variables.

As discussed, the dataset was prepared using survey data from NHANES; the modules used here are demographic, examination, laboratory, and questionnaire data. An example of mathematical description of the dataset using some of the features as an example has been given in Table 2, and data distribution considering CHD/no-CHD was depicted in Figure 8. After removing the missing and outlier values, the total number of instances was 40,713. Since the sequence number, family income, height, insurance, etc., information did not have diagnostic significance, those columns were dropped from the primary dataset. In addition, not all the features or column variables were numeric data, such as Gender, Diabetes, Vigorous-work, Moderate-work, etc., so those data were modified from categorical to numeric by dividing each of the columns into multiple columns. After dropping the mentioned columns and distributing the categorical columns, the final dataset contained 40,713 rows and 47 columns, including the class variable (CHD). An example of a correlation matrix has been depicted as a heatmap using a subset (15) of randomly chosen features chosen along with the target variable in Figure 9.

### 3.2. Feature Selection Process

The chosen feature selection method for this study was the ensemble method. After using mRMR from filtering, RFE from the wrapper, and elastic-net from the embedded strategy, the outcomes of the feature ranking or subset were aggregated using the ensemble method. The technique used here was ‘mRMR,’ a filter-based method. The specific reason to pick the mRMR is that it can choose relevant features while eliminating irrelevant ones. In terms of correlation, it can be explained that the selected features are highly correlated with the class variable but show a low correlation among them [112]. The features are selected individually using a function that ensures relevance and redundancy. Two standard objective functions used for mRMR are the mutual information difference criterion (MID) and the mutual information quotient criterion (MIQ). The MIQ criterion measures the ratio of the mutual information between two variables to the average mutual information of all possible pairs of variables. It can identify pairs of variables with relatively high mutual information compared to other pairs. In this study, the second objective function type (MIQ) has been applied. Suppose there are a total of n features. In that case, *B* is denoted as a class variable, and *m* is the number of selected features. For a given feature *A_i_*, where ‘*i*’ varies between 1 and n, the feature importance can be expressed as follows [113]:

(1) $$f^{mRMR}\left(A_{i}\right)=I\left(B,A_{i}\right)-\frac{1}{m}\sum_{A_{m}€m}I\left(A_{m},A_{i}\right)$$

*I*(*B*, *A*) denotes the mutual information between *B* and *A*. The two variants of standard *mRMR* use objective functions as follows [113]:

(2) $$f^{MID}\left(A_{i}\right)=I\left(B,A_{i}\right)-\frac{1}{m}\sum_{A_{m}€m}I\left(A_{m},A_{i}\right)$$

(3) $$f^{mRMR}\left(A_{i}\right)=I\left(B,A_{i}\right)/\frac{1}{m}\sum_{A_{m}€m}I\left(A_{m},A_{i}\right)$$

Since mRMR results in ranking features, the MIQ objective functions were implemented using Python to find the ranking of the top 30 features (except the categorical features). 

**Figure 10:**
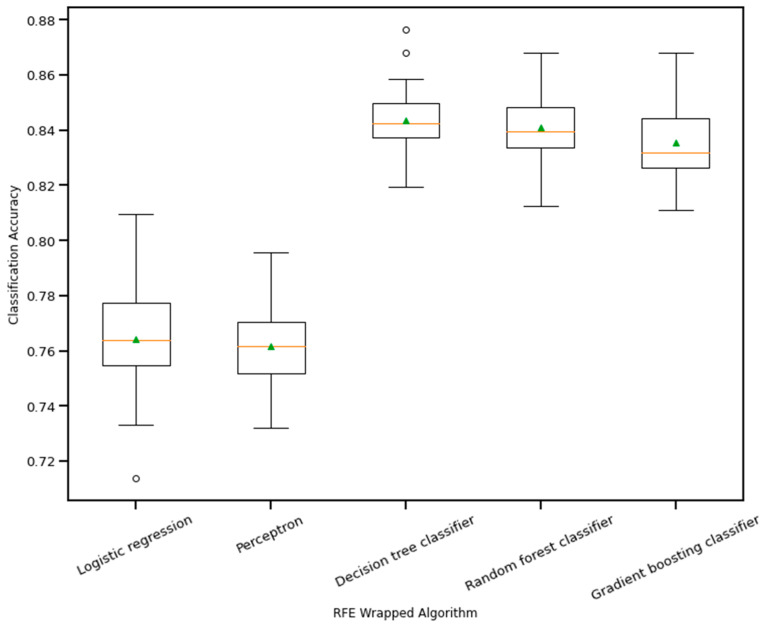
Boxplot of Recursive feature elimination wrapped algorithm vs. classification accuracy. The green triangle and the orange line indicate mean and median value of model accuracy respectively.

**Figure 11:**
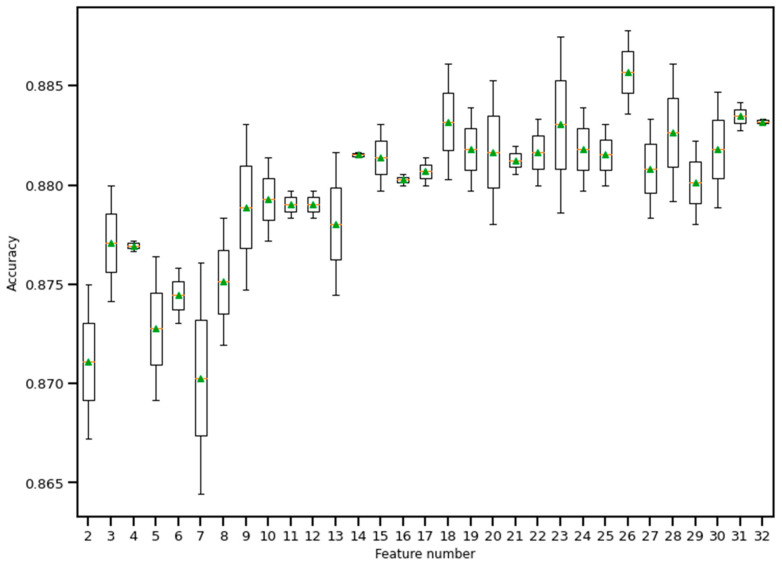
Boxplot of Recursive feature elimination technique for feature selection. Here, a random forest classifier has been used to find the accuracy as features keep entering into the selection set. The green triangle and the orange line indicate mean and median value of model accuracy respectively.

RFE is a wrapper-based feature selection technique. It uses a chosen classifier to check the performance of a different subset of features, and, based on chosen metrics, the final subset of features is finalized. Thus, in RFE, there are two primary options to be selected: one is the number of selected features, and the other is the algorithm that will be used to choose the features. As shown in Figure 10, five different algorithms were used in the core RFE using the dataset finalized in Section 3.1, excluding the categorical features. The five algorithms were logistic regression, decision tree classifier, perceptron, random forest classifier, and gradient boosting classifier. As well, using a random forest classifier, the effect on accuracy after adding each feature was monitored, as shown in Figure 11. The aim function for the sum of squared error is as follows [114]:

(4) $$minimize\;\{SSE=\sum_{i=1}^{n}{{[y}_{i}-y_{i}(mean)]}^{2}={\left|\left|y-X\beta_{est}\right|\right|}^{2}\}$$

Here, *n* is the observation number, *y* is the predicting variable, the predictor variable is *X*, the error is assumed to be normally distributed, and its variance is σ^2^, since *β* is unknown and can be measured from the sample data. The difference between the actual and estimated *β* is the bias. The ridge-type regularization technique adds a penalty in the aim function to adjust the value of the coefficients as follows [114]:

(5) $$minimize\;\{SSE+lambda\sum_{j=1}^{p}\beta_{j}^{2}\}$$

By changing the value of the ‘lambda’, the penalty parameter, ridge regularization tends to push some of the feature values close to zero (but not actually zero). As such, this way, the number of features does not reduce, but the impact of some of the features diminishes. On the other hand, lasso regularization is a modified version of the ridge method that penalizes the sum of the absolute value of the coefficients. However, lasso removes features by pushing the coefficient value to zero.

Choosing the appropriate value of lambda is essential to keep the number of features optimum (not too high or not too low). Thus, in this study, the implemented one was elasticnet. Elasticnet’s aim function contains another parameter as *α*; the value of this decides how much the elasticnet moves closer to either ridge or lasso. The following is the aim function of elasticnet [114]:

(6) $$minimize\;\{SSE+{lambda}_{2}\times \alpha \times \sum_{j=1}^{p}\left|\beta_{j}\right|+{lambda}_{1}\times \frac{1-\alpha }{2}\times \sum_{j=1}^{p}\beta_{j}^{2}\}$$

The loss function for quadratic regression is firmly convex, meaning it has a unique minimum. Linear regression often results in a model with low bias but high variance, which means that it may not generalize well to new data. We can add some bias to the model by introducing regularization to reduce the variance. One way to do this is by combining ridge and lasso regression. Both add penalties to the loss function to constrain the model complexity. This can help improve the overall performance of the model. The elasticnet module from Python was used to produce the selected feature set. The aggregation method combined the features from the three feature selection algorithms, and the final number of numeric features was 24. The categorical features were added after that. Table 3 contains the list of final features after implementing feature selection algorithms. 

### 3.3. The 1D CNN Model

The one-dimensional convolutional neural network (1D CNN) started with the input from the finalized selected feature list, consisting of numerical and encoded categorical features. Each categorical feature was divided into several features by one hot encoding, making the total feature number 40. The input went through a dense layer to mix the features well, so that the issue of the chronology of features did not make any difference. The number of nodes in the first dense layer was 600, with an activation layer as ‘relu’ and dropout as 0.3. As discussed in the previous section about removing the dependency on the chronology of features, the dense layer output was reshaped into 20 channels, 1 × 30 shape.

**Figure 12:**
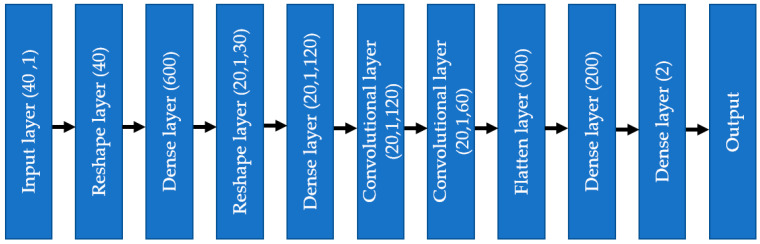
One-dimensional convolutional neural network diagram.

As depicted in Figure 12, the input data flowed through different layers: reshaped, dense, convolutional, flattened, and dense, to reach the output. Adam optimizer with a learning rate = 0.001 was used, and the selected loss function was “categorical_crossentropy”. The chosen metric for the model was ‘accuracy.’ The whole model was trained and tested using jupyter notebook in Google Collab on a laptop with the following configuration: Intel core i7 1065G7 CPU @ 1.30 GHz, 24 GB RAM (8 + 16) DDR4, no GPU (the benefit of using 1D CNN compared to 2D CNN).

**Figure 13:**
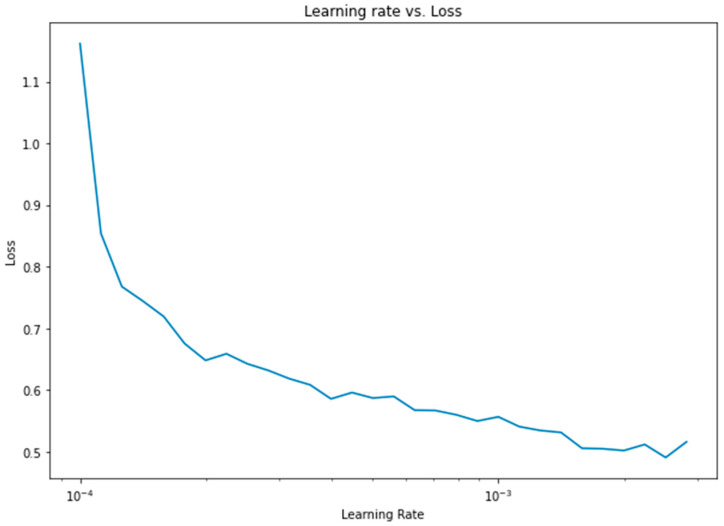
Learning rate vs. loss diagram for 1D CNN.

Apart from the weight assignment to class variables, another important optimization was the learning rate. To find the optimum learning rate, a semilog-based plot was created, using the data from the learning rate range and loss parameters, as shown in Figure 13. 

To evaluate the performance of the 1D CNN, some other machine learning algorithms were used for the same dataset to compare them with the proposed method. The ML algorithms used a\were ANN with two dense layers, logistic regression, support vector machine, AdaBoost classifier, and random forest classifier. 

## 4. Results

This section discusses the results from the data analysis section. The discussion starts with the 1D CNN architecture model results, followed by a comparison with other models. A confusion matrix was depicted to elaborate on the performance of class variables. Also, the weight assignment process and performance metrics, such as accuracy and loss in terms of epochs, were discussed. Finally, four more types of heart disease were used for similar studies, and the results were also summarized.

**Figure 14:**
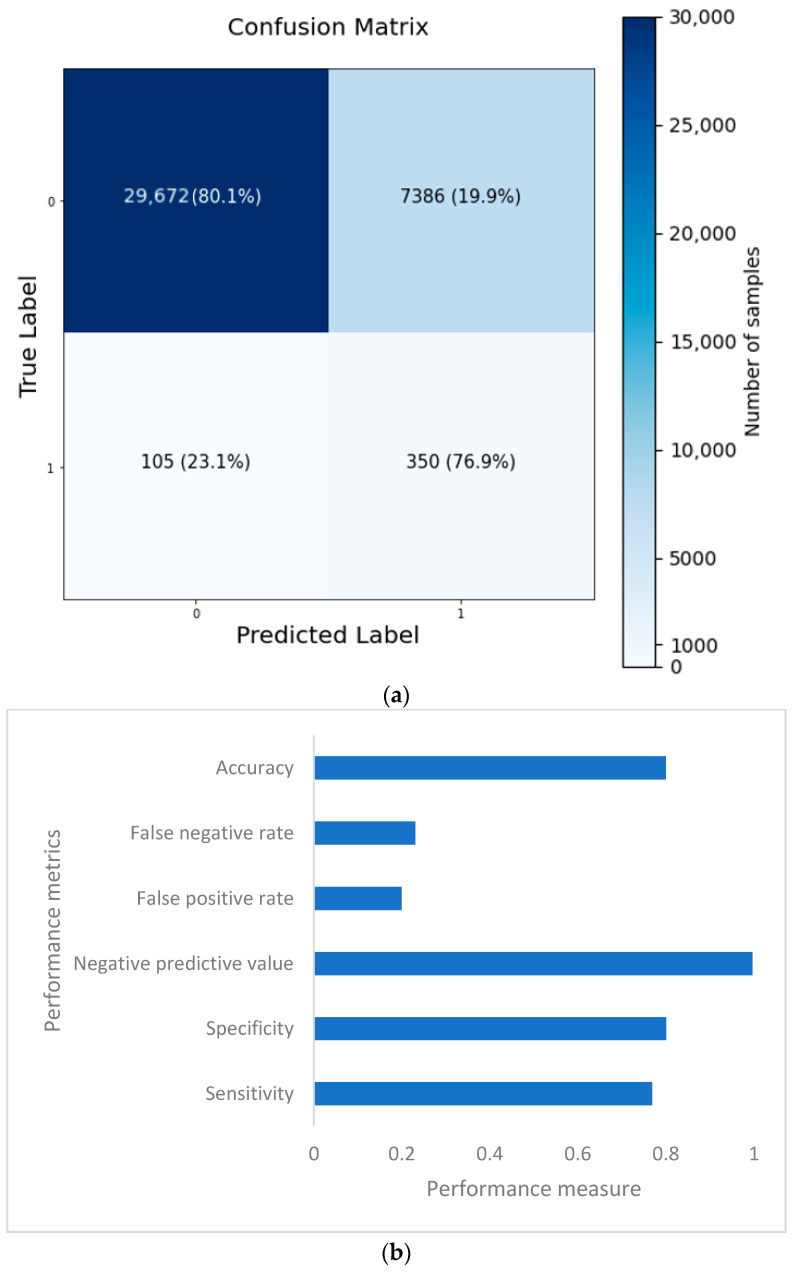
Confusion matrix (**a**) and performance metrics (**b**) for the 1D CNN model.

**Figure 15:**
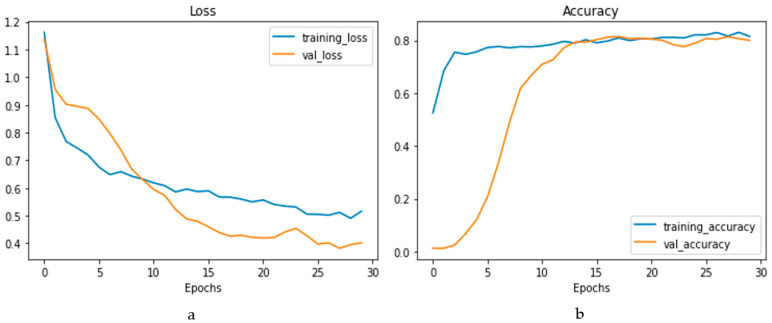
Loss vs. epochs (**a**) and accuracy vs. epochs (**b**) graph for the 1D CNN model.

The confusion matrix is depicted in Figure 14a, and the performance metrics are plotted in Figure 14b. It is evident that, despite a significant imbalance in the dataset, the implementation of 1D CNN brought forth improved accuracy. Figure 15a shows the loss vs. epochs graph. Although the loss was initially high (approximately 25 epochs), the loss steadied around 0.4. Also, in Figure 15b, the accuracy of the model vs. epochs graph is depicted. The accuracy of the validation set started slow. However, at 15 epochs, it was close to the training accuracy and kept increasing slowly to 30 epochs. 

**Figure 16:**
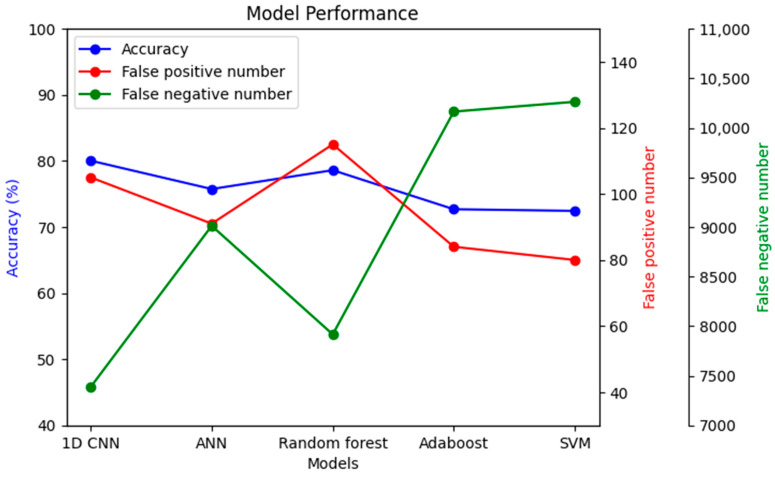
Performance comparison among different models with the same dataset.

To understand the significance of introducing a convolutional layer (CNN) into the model, another model was prepared with only one convolutional layer. The results showed that the false negative results increased from 23.1% to 49.2%. Moreover, the same dataset was used with a few other models to compare performance, as shown in Figure 16. 

The imbalanced dataset’s main challenge was that many models’ accuracy may be high. However, in nearly all cases, the accuracy came as a sacrifice, also causing a very high false negative number. As seen in Figure 16, the false negative number is the lowest in the case of 1D CNN, while accuracy simultaneously remained the highest. Alternatively, the false positive number might be improved in the random forest classifier compared to 1D CNN, but with lower accuracy and a higher false negative number. 

Similar to the CHD dataset, four more datasets were prepared from NHANES survey data using similar strategies for heart attack, angina pectoris, congestive heart disease, and stroke. Elasticnet was applied for all of them to obtain a feature list for model training. The general 1D CNN, which was used for this four heart condition dataset, consisted of the following steps. First, the reshape layer was applied to transform the flattened feature set into multiple one-dimensional data segments. After that, one dense layer, one convolutional layer, and another dense layer were applied to mix the effect of features and remove the effect of the chronology of features in the dataset. The class weight was 1:5, and the learning rate used was 0.001. 

**Figure 17:**
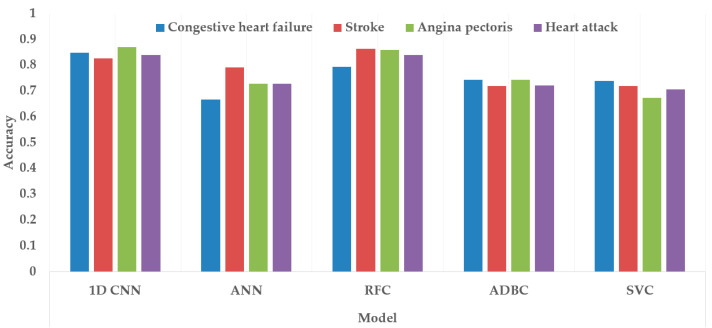
Comparing accuracy among different class variables (heart conditions) using different classification models.

**Figure 18:**
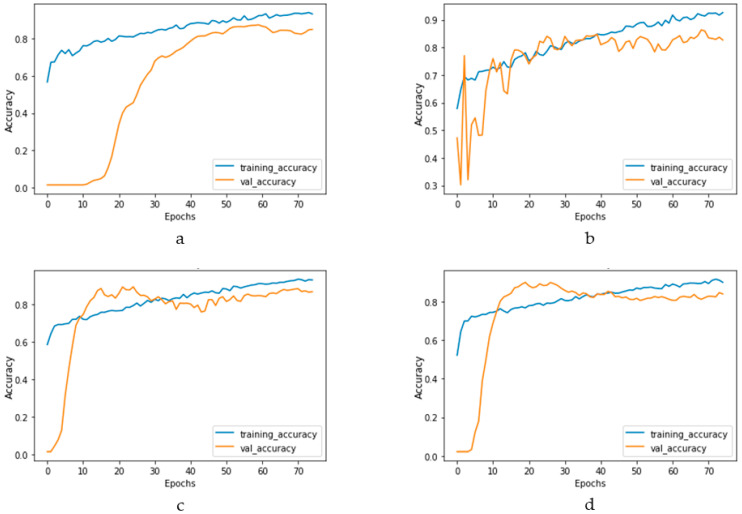
Training and validation accuracy vs. epochs graph for (**a**) congestive heart failure, (**b**) stroke, (**c**) angina pectoris, (**d**) heart attack analysis.

**Figure 19:**
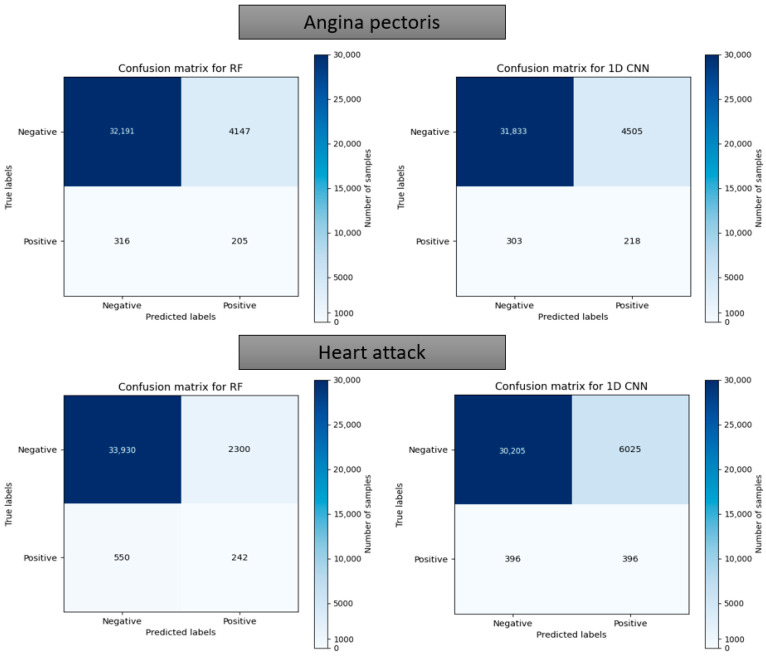
Confusion matrix using the random forest and 1D CNN in case of “Angina pectoris” and “Heart attack”.

Figure 17 and Figure 18 depict the model performance of four datasets (congestive heart failure, stroke, angina pectoris and heart attack), comparing the proposed 1D CNN with several other ML models such as ANN, RFC, ADBC, SVC, etc. Using Keras, an artificial neural network (ANN) with three hidden layers (64, 128, and 256 units) was built throughout this training procedure. The Adam optimizer with a learning rate of 0.001 was used to optimize the model. The optimization aim was to reduce the categorical cross-entropy loss function while increasing the accuracy metric. Dropout regularization occurred at a rate of 0.2 after each hidden layer to prevent overfitting. After each dense layer, batch normalization was used to increase training stability and generalization performance. The use of class weights was one significant component of this training technique. By giving class 1 a larger weight (2.2 times) than class 0, the model prioritized properly forecasting instances of the minority class, addressing class imbalance difficulties. The random forest classifier was used to optimize its hyperparameters. GridSearchCV was used to perform a grid search using several estimators (10, 30, 50, 100, 130, 160, 200) and maximum depths (2, 3). The classifier was trained using balanced class weights on the original training data. Following the grid search, the improved random forest classifier was built using the best parameters discovered during the search. After that, the improved classifier was trained on the original training data. A Support Vector Classifier (SVC) was trained in this approach, with the hyperparameters C = 0.05, gamma = 1/41, kernel = ‘rbf’, and class_weight = ‘balanced’. First, the accuracy using 1D CNN model for the four conditions is shown in Figure 18. The accuracy of 1D CNN was consistent. Notably, the accuracy in the random forest classifier was higher in two heart conditions than in 1D CNN. However, when it was investigated, it was found that, in both cases, the false negative number was significantly higher, deeming the overall performance of 1D CNN better for heart disease, as shown in Figure 17 and Figure 19. Additionally, in Figure 19, the loss and accuracy of the model (1D CNN) concerning epochs are depicted. 

## 5. Discussion

In nearly all the studies performed for identifying symptoms of heart disease using artificial intelligence, the datasets used were smaller. Features were not created to use them for machine learning, as several of the features within those limited numbers needed to be more relevant. That was why bringing forward a dataset with many relevant features and a significantly higher number of samples was essential. This study used undersampling and weight class assignments to mitigate the data imbalance issue. The critical change at the beginning of the 1D CNN model was the reshaping from the initial dense layer. The purpose of this reshaping was to allow the model to perform well, irrespective of the chronological order of the features. Thus, the input layer was reshaped and fed into a dense layer, which mixed the effect of a different subset of features using a convolutional layer.

Based on the evaluation measures, the suggested 1D CNN model for coronary heart disease performed well. The model had a sensitivity of 0.7692, suggesting its ability to detect affirmative cases properly. The model’s specificity of 0.8007 demonstrates its ability to reliably detect negative situations. The model has a high likelihood of properly recognizing real negative instances, with a negative predictive value of 0.9965. The model’s accuracy of 0.8003 indicates that it attained an overall good prediction rate. The false positive rate of 0.1993 and false negative rate of 0.2308 reveal the trade-off between improperly identifying positive and negative cases, respectively, and suggest more areas for improvement in the model. A comparison of several categorization algorithms helepd to understand the impact of introducing 1D CNN layer while predicting coronary heart disease (CHD) using NHANES data. In nearly all cases, the accuracy came as a sacrifice to a very high false negative number. Each model’s performance was evaluated using a variety of criteria, including accuracy, sensitivity, specificity, negative predictive value, false positive rate, and false negative rate. In terms of overall accuracy, the 1D CNN model scored the maximum accuracy of 80.03%. It outperformed ANN (75.74%), Random Forest Classifier (78.59%), Adaboost Classifier (72.68%), and Support Vector Machine (72.43%). In terms of sensitivity, which assesses the model’s capacity to correctly identify positive cases, the 1D CNN model had a sensitivity of 0.7692, suggesting its ability to effectively identify real positive cases. The Random Forest Classifier had a sensitivity of 0.7859, whereas the ANN model had a slightly lower sensitivity of 0.7574. The sensitivities of the Adaboost Classifier and Support Vector Machine were 0.7268 and 0.7243, respectively. Specificity values, which represent a model’s ability to properly detect negative situations, were comparable among the models. The 1D CNN model had a specificity of 0.8007, whereas the Random Forest Classifier had a specificity of 0.7993. Looking at the false positive and false negative values, the 1D CNN model had 105 false positives and 7386 false negatives. Although it had the best accuracy, it also had the largest false negative count when compared to the other models. The ANN model, on the other hand, had a somewhat lower accuracy of 75.74%, with 91 false positives and 9011 false negatives. The Random Forest Classifier was 78.59% accurate, with 115 false positives and 7916 false negatives. The accuracy of the Adaboost Classifier was 72.68%, with 84 false positives and 10,164 false negatives. Finally, the Support Vector Machine model was 72.43% accurate, with 80 false positives and 10,263 false negatives. The examination of the suggested models for four illnesses, namely, congestive heart failure, stroke, angina pectoris, and heart attack, gives intriguing insights into the performance of the 1D CNN model. The 1D CNN outperformed the other models in terms of congestive heart failure prediction, with an accuracy of 0.8488. Similarly, the 1D CNN obtained an accuracy of 0.8266 in stroke prediction, suggesting its ability to effectively detect stroke instances. Furthermore, the 1D CNN outperformed competitors in angina pectoris and heart attack prediction, with accuracies of 0.8696 and 0.8393, respectively. Major findings emphasize the superiority of the 1D CNN model over the other models in effectively predicting major cardiovascular illnesses. These findings imply that the proposed model offers great promise for improving different disease prediction accuracy in the field of cardiovascular health. Further study and improvement of the model might lead to enhanced diagnostic skills and patient outcomes. 

There were several limitations and constraints in the study. Although the number of features available in this study was not only higher in number compared to contemporary studies but also the features were more relevant, the model would be better trained if more information were available. This is because the detection or diagnosis does not depend on a single parameter, such as arrhythmia, whereas diagnosis depends on the number of heartbeats per minute. The heart diseases mentioned in this study are more complex, with symptoms and effects spread over many parts of the body and features, for example, stroke originates in the narrowing of blood vessels due to higher blood pressure or the bursting of blood vessels in the brain. Acquiring stroke symptoms is not as simple as counting one or a few vital parameters. Instead, many laboratory parameters, the heart’s electrical and mechanical activities, the situation in blood vessels, the overall physiological situation, etc., are involved. That is why, even though the NHANES survey did a better job compared to other critical data surveys about the health condition, it is worth noting that more specific information about heart health would help build a better prediction model. As such, a significant limitation of this study is that the feature list had to be accumulated from the NHANES survey data. There must be an initiative with global standards to collect patient data relevant to building predictive models to resolve this issue. Another limitation of this study is the need for more interpretability. The black-box nature of the deep learning model makes it difficult to explain how the complex network architecture and non-linear operations arrive at a prediction. To bring all stakeholders (technical and non-technical) related to the solution into confidence, the decision-making process needs to be more transparent. The interpretability of the deep learning model is an active area of research right now. 

## 6. Conclusions

Heart disease is one of the fatal chronic diseases which becomes very difficult to cure if detected at later stages. On the other hand, proper treatment can be given if diagnosed early. In fact, in some cases, a lifestyle change may improve the health condition. Early detection also positively impacts the patient’s family regarding financial aspects compared to later diagnosis. This paper proposes using a larger dataset, ensemble feature selection algorithm, and modified 1D CNN to classify clinical parameters into different heart disease classifications. Through experiments and evaluations using the dataset acquired from NHANES, our modified 1D CNN model achieved higher accuracy for overall heart disease detection than other ML techniques. Furthermore, the results indicated that our approach withstood the challenge of an unbalanced dataset compared to other techniques, producing the lowest number of false negatives. The confusion matrix confirmed that all four elements were above 75%, even with the imbalanced dataset, with the most prominent effect for the false negative value. The loss and accuracy curve against number of epochs showed that, around 30 epochs, the loss and accuracy become steady during training and validation. The 1D CNN showed better accuracy, false positives, and negative numbers than other ML methods. The values where a few of the ML methods performed similarly proved one-sided. In those cases, the higher performance came as a higher contribution from the majority class (non-CHD). The significant limitations were imbalanced data, the lack of explain ability (a general consequence of a deep learning model), and the requirement for more extensive research in the other four heart conditions. In the future, steps will be taken to solve or mitigate those limitations and make the model robust and accurate for all heart health conditions. 

## Figures and Tables

**Table 1:** Data documentation of blood pressure, an example from NHANES [73].

Variable Name	Description	Target	Code Value	Code Value Description
SEQN	Respondent sequence number	All age groups	NA.	NA
PEASCCT1	Blood Pressure Comment	All age groups	1, 2, 3	Safety exclusion, SP refusal, time constraint (Age was divided into three groups named 1, 2, and 3)
BPXCHR	60 s HR (30 s HR × 2)	0–7 years	60–180 bits per minute	Range of values in heart bit per minute
BPAARM	Arm selected	8 years and above	1, 2, 8	Right, left, could not obtain (Correspond to 1, 2, and 8)
BPACSZ	Coded cuff size	8 years and above	1, 2, 3, 4, 5	Infant, Child, Adult, Large, Thigh (each number in code value represents one age group)
BPXPLS	60 s pulse (30 s pulses × 2)	8 years and above	34–136	Range of number of pulses in 60 s
BPXPULS	Pulse regular or irregular?	All age groups	1, 2	Regular, irregular (1—regular, 2—irregular)
BPXPTY	Pulse type	8 years and above	1, 2, 8	Radial (1), brachial (2), could not obtain (8)
BPXML1	MIL: maximum inflation levels (mmHg)	8 years and above	100–260	Range of values of blood pressure in mmHg
BPXSY1	Systolic: Blood pres (1st rdg) mmHg	8 years and above	72–228	Range of values of blood pressure in mmHg
BPXDI1	Diastolic: Blood pres (1st rdg) mmHg	8 years and above	0–136	Range of values of blood pressure in mmHg
BPAEN1	Enhancement used 1st rdg	8 years and above	1, 2, 8	Yes (1), no (2), could not obtain (8)
BPXSY2	Systolic: Blood pres (2nd rdg) mmHg	8 years and above	72–236	Range of values of blood pressure in mmHg
BPXDI2	Diastolic: Blood pres (2nd rdg) mmHg	8 years and above	0–136	Range of values of blood pressure in mmHg
BPAEN2	Enhancement used 2nd rdg	8 years and above	1, 2, 8	Yes (1), no (2), could not obtain (8)
BPXSY3	Systolic: Blood pres (3rd rdg) mmHg	8 years and above	72–238	Range of values of blood pressure in mmHg
BPXDI3	Diastolic: Blood pres (3rd rdg) mmHg	8 years and above	0–134	Range of values of blood pressure in mmHg
BPAEN3	Enhancement used third reading	8 years and above	1, 2, 8	Yes (1), no (2), could not obtain (8)
BPXSY4	Systolic: Blood pres (4th rdg) mmHg	8 years and above	72–234	Range of values of blood pressure in mmHg
BPXDI4	Diastolic: Blood pres (4th rdg) mmHg	8 years and above	0–118	Range of values of blood pressure in mmHg
BPAEN4	Enhancement used 4th rdg	8 years and above	1, 2, 8	Yes (1), no (2), could not obtain (8)

**Table 2:** A mathematical description of the dataset with some features as an example.

Parameter	Age (Years)	Systolic (mmHg)	Diastolic (mmHg)	Weight (kg)	Height (cm)	Cholesterol (mg/dL)	Creatinine (mg/dL)	Glucose (mg/dL)	Protein (mg/dL)
mean	48.98	124.18	71.00	81.26	167.34	5.06	78.36	5.60	72.00
STD	17.83	19.15	11.72	20.84	10.11	1.08	35.34	2.04	4.89
Min	20.00	66.00	40.00	32.30	129.70	0.16	17.70	1.05	47.00
25th percentiles	34.00	111.00	64.00	66.60	160.00	4.29	61.88	4.72	69.00
50th percentiles	48.00	122.00	71.00	78.40	167.00	4.97	73.37	5.11	72.00
75th percentiles	63.00	134.00	78.00	92.40	174.60	5.71	88.40	5.66	75.00
Max	85.00	270.00	132.00	223.00	204.50	14.61	946.76	34.75	113.00

**Table 3:** Finalized list of features after feature selection algorithms.

No	Feature	Feature Type	Description
1	Age	Numerical	Age of participant (years)
2	Systolic	Numerical	Systolic blood pressure (mmHg)
3	Diastolic	Numerical	Diastolic blood pressure (mmHg)
4	Weight	Numerical	Weight of participant (kg)
5	White-Blood-Cells	Numerical	White blood cell count (1000 cells/μL)
6	Lymphocyte	Numerical	Lymphocyte percent (%)
7	Monocyte	Numerical	Monocyte percent (%)
8	Red-Blood-Cells	Numerical	Red blood cell count (million cells/μL)
9	Platelet-count	Numerical	Platelet count (1000 cells/μL)
10	Red-Cell-Distribution-Width	Numerical	Red cell distribution width (%)
11	Albumin	Numerical	Albumin, urine (mg/L)
12	ALP	Numerical	Alkaline Phosphatase (IU/L)
13	ALT	Numerical	Alanine Aminotransferase (IU/L)
14	Cholesterol	Numerical	Cholesterol (mg/dL)
15	Creatinine	Numerical	Creatinine (mg/dL)
16	Glucose	Numerical	Glucose, serum (mg/dL)
17	GGT	Numerical	Gamma-glutamyl transferase (U/L)
18	Iron	Numerical	Iron, refrigerated serum (μg/dL)
19	LDH	Numerical	Lactate dehydrogenase (IU/L)
20	Triglycerides	Numerical	Triglycerides, refrigerated (mg/dL)
21	Uric.Acid	Numerical	Uric acid (mg/dL)
22	Total-Cholesterol	Numerical	Total Cholesterol (mg/dL)
23	HDL	Numerical	Direct HDL-Cholesterol (mg/dL)
24	Glycohemoglobin	Numerical	Glycohemoglobin (%)
25	Gender	Categorical	Gender of the participant
26	Diabetes	Categorical	Diagnosed with Diabetes
27	Blood rel Diabetes	Categorical	Does blood relative have Diabetes
28	Blood rel stroke	Categorical	Does a blood relative have a stroke
29	Vigorous work	Categorical	Vigorous work activity
30	Moderate work	Categorical	Moderate work activity

## Data Availability

Publicly available datasets were analyzed in this study. This data can be found here: [https://www.cdc.gov/nchs/nhanes/index.htm] (accessed on 31 May 2023).
